# *MSH6 *and *PMS2 *mutation positive Australian Lynch syndrome families: novel mutations, cancer risk and age of diagnosis of colorectal cancer

**DOI:** 10.1186/1897-4287-8-5

**Published:** 2010-05-21

**Authors:** Bente A Talseth-Palmer, Mary McPhillips, Claire Groombridge, Allan Spigelman, Rodney J Scott

**Affiliations:** 1 School of Biomedical Sciences and Pharmacy, University of Newcastle, NSW 2308, Australia; 2 Hunter Medical Research Institute, John Hunter Hospital, Newcastle, NSW 2305, Australia; 3 Hunter Area Pathology Service, Hunter New England Area Health, NSW 2305, Australia; 4 Hunter Family Cancer Service, Hunter New England Area Health, NSW 2305, Australia; 5 University of NSW, St Vincent's Hospital Clinical School, Sydney, NSW 2010, Australia

## Abstract

**Background:**

Approximately 10% of Lynch syndrome families have a mutation in *MSH6 *and fewer families have a mutation in *PMS2*. It is assumed that the cancer incidence is the same in families with mutations in *MSH6 *as in families with mutations in *MLH1/MSH2 *but that the disease tends to occur later in life, little is known about families with *PMS2 *mutations. This study reports on our findings on mutation type, cancer risk and age of diagnosis in *MSH6 *and *PMS2 *families.

**Methods:**

A total of 78 participants (from 29 families) with a mutation in *MSH6 *and 7 participants (from 6 families) with a mutation in *PMS2 *were included in the current study. A database of de-identified patient information was analysed to extract all relevant information such as mutation type, cancer incidence, age of diagnosis and cancer type in this Lynch syndrome cohort. Cumulative lifetime risk was calculated utilising Kaplan-Meier survival analysis.

**Results:**

*MSH6 *and *PMS2 *mutations represent 10.3% and 1.9%, respectively, of the pathogenic mutations in our Australian Lynch syndrome families. We identified 26 different *MSH6 *and 4 different *PMS2 *mutations in the 35 families studied. We report 15 novel *MSH6 *and 1 novel *PMS2 *mutations. The estimated cumulative risk of CRC at age 70 years was 61% (similar in males and females) and 65% for endometrial cancer in MSH6 mutation carriers. The risk of developing CRC is different between males and females at age 50 years, which is 34% for males and 21% for females.

**Conclusion:**

Novel *MSH6 *and *PMS2 *mutations are being reported and submitted to the current databases for identified Lynch syndrome mutations. Our data provides additional information to add to the genotype-phenotype spectrum for both *MSH6 *and *PMS2 *mutations.

## Introduction

Hereditary nonpolyposis colorectal cancer (HNPCC)/Lynch syndrome (MIM 120435) accounts for approximately 2 percent of all diagnosed colorectal cancers (CRC) [1]. Lynch syndrome is an autosomal dominantly inherited cancer syndrome characterised by early onset epithelial cancers. Patients with Lynch syndrome have an increased risk of developing malignancies during their lifetime, at a mean age of disease onset that is significantly lower than that observed in the general population. In addition to the high risk of developing CRC, Lynch syndrome patients are also at risk of developing malignancies in a variety of organs that include the uterus, small bowel, stomach, ovary, bladder, pancreas and the urinary tract [2,3]. A breakdown in the fidelity of DNA mismatch repair has been shown to be the basis of the disease. At present four genes encoding proteins that are integrally involved in DNA mismatch repair (MMR) have been clearly associated with Lynch syndrome and these are *MLH1 *(MIM 120436), *MSH2 *(MIM 609309), *MSH6 *(MIM 600678) and *PMS2 *(MIM 600259) [4-7]. MMR provides several genetic stabilisation functions; it corrects DNA biosynthesis errors, ensures the fidelity of genetic recombination and participates in the earliest steps of cell cycle checkpoint/control and apoptotic responses [8]. MMR gene defects increase the risk of malignant transformation of cells, which ultimately result in the disruption of one or several genes associated with epithelial integrity [8,9].

Genetic testing of *MLH1 *and *MSH2 *for Lynch Syndrome has been available for over a decade and during this time significant advances in the technologies used for diagnosis have occurred. Together with improvements in technology the ability to rapidly screen additional genes associated with Lynch syndrome, *MSH6 *and *PMS2*, has become available. The MMR genes *MSH6 *and *PMS2 *have been shown to interact with *MSH2 *and *MLH1*, respectively [9,10]. Impediments to screening these two genes for mutations have been the high cost of testing and the presence of pseudogenes in *PMS2 *[11-13]. Notwithstanding, some information is available with respect to the frequency of *MSH6 *and *PMS2 *mutations but there is relatively limited information available regarding the spectrum of disease, especially in Australian Lynch syndrome families harbouring deleterious changes in one of these two genes.

Approximately 10 percent of Lynch syndrome families have a mutation in *MSH6 *and fewer families have a mutation in *PMS2 *[14]. It is assumed that the cancer incidence is the same in families with mutations in *MSH6 *as in families with mutations in *MLH1 *and *MSH2 *but that disease tends to occur later in life as a result of the partial compensation provided by *MSH3 *in MMR [15]. *PMS2 *mutations lead to an attenuated phenotype with weaker family history and older ages of disease onset [15]. Phenotype information for *MSH6 *and *PMS2 *mutation carriers is therefore of great interest for the recognition of Lynch syndrome and the formulation of sufficient surveillance schemes.

In the current study we report on our findings on mutation type, cancer risk and age of diagnosis in 29 families (78 participants) with a mutation in *MSH6 *and 6 families (7 participants) with a mutation in *PMS2*.

## Materials and methods

All the participants selected for this study had previously been diagnosed with Lynch syndrome and harboured a mutation in either *MSH6 *or *PMS2*. The study was conducted in accordance with the Declaration of Helsinki. Approval for the study was obtained from Hunter New England Human Research Ethics Committee and the University of Newcastle Human Research Ethics Committee. Written, informed consent was obtained from all participants.

Since 1997, samples have been tested for Lynch syndrome/HNPCC at the Division of Genetics, Hunter Area Pathology Service in Newcastle, New South Wales, Australia. Information from all the samples collected between 1997 and 2008 has been placed into a database. The families were referred for genetic testing due to a clinical diagnosis of HNPCC according to the Amsterdam II criteria/Bethesda criteria or due to the tumour displaying microsatellite instability (MSI) or immunohistochemistry (IHC) demonstrating loss of *MSH6/PMS2 *expression.

A total of 78 participants (from 29 Caucasian families) with a mutation in *MSH6 *and 7 participants (from 6 Caucasian families) with a mutation in *PMS2 *were included in the current study. A database of de-identified patient information was analysed to extract all relevant information such as mutation type, cancer risk, age of diagnosis and cancer type in this patient cohort. Of the 29 *MSH6 *mutation positive families, 16 fulfilled the Amsterdam II criteria and 1 fulfilled the Bethesda criteria, while 6 did not fulfil the guidelines for either (5 cases had loss of staining of MSH6, while 1 was MSI-High) and in 5 families the status was unknown (no pedigree/uninformative pedigree). Of the 6 *PMS2 *mutation positive families, 4 fulfilled the Amsterdam II criteria, while 2 families did not due to lack of family history of cancer or uninformative pedigree.

The diagnosis of the cancers in the mutation positive participants was confirmed in histopathological reports, while information of cancers in family members with unknown mutation status is collected from the family pedigrees.

Cumulative lifetime risk for MSH6 mutation carriers was calculated using Kaplan Meier survival analysis, and was determined on the basis of CRC being the 1^st ^primary tumour against cancer free individuals and endometrial cancer being the 1^st ^primary tumour against cancer free females. The observation time for the different cases was from birth until first cancer diagnosis or last follow-up appointment.

## Results

### *MSH6 *families

We identified 26 different *MSH6 *mutations in the 29 probands; a list of the mutations is shown in Table 1. Eleven of the identified *MSH6 *mutations have been reported before [16-23] and fifteen *MSH6 *mutations are novel mutations (six of these have been posted on the LOVD database and the remaining nine will be submitted). All mutations are considered causative and predictive testing has been offered to family members. Table 1 also lists available immunohistochemistry (IHC) and microsatellite instability (MSI) results from the participant's tumours. IHC results were available in 20 of the 29 families; the tumour of 16 probands showed lack of staining (-ve) for *MSH6*, 2 tumours were -ve *MSH2 *but positive (+ve) for *MSH6*, 1 tumour showed isolated loss of *MSH6 *while 1 was uninformative for *MSH6 *but +ve for *MLH1*, *MSH2 *and *PMS2*. MSI results were available from 6 probands, all displaying MSI-High (unstable), 4 of them belonging to the -ve *MSH6 *group.

**Table 1:** List of the *MSH6 *probands, IHC/MSI results and mutation information including; exon, nucleotide change, consequence of mutation and references.

Family ID	IHC/MSI results	Exon	Nucleotide Change	Consequence of Mutation	LOVD database ID
MSH6_10	-veMSH2, +veMSH6	1	c.1_457del	Deletion	DB-ID: MSH6_00001

MSH6_8	Not available	2	c.458_627del	Truncating	DB-ID: MSH6_00336

MSH6_5	-ve MSH6	3	c.458_657del	Exon deletion	Not previously reported

MSH6_29	Not available	3	c.620del	Frameshift	Not previously reported

MSH6_7	+ve MLH1, MSH2, PMS2. MSH6; uninformative MSI-High	4	c.674insTG	Frameshift/Stop	Not previously reported

MSH6_19	-ve MSH6	4	c.710delG	Frameshift/Stop	DB-ID: MSH6_00702*

MSH6_17	Not available	4	c.723dupT	Nonsense substitution/Stop	Not previously reported

MSH6_23	-ve MSH6	4	c.1404_1405delTC	Truncating	Not previously reported

MSH6_4	-ve MSH6 MSI-High	4	c.2150_ 2153delTCAG	Frameshift	DB-ID: MSH6_00175

MSH6_27	MSH6; isolated loss	4	c.2348_2349del	Truncating	DB-ID: MSH6_00442

MSH6_6	Not available	4	c.2765delG	Frameshift	DB-ID:MSH6_00703*

MSH6_1	Not available	4	c.2535dupT	Frameshift/Stop	DB-ID: MSH6_00701*

MSH6_20	-ve MSH6 MSI-High	4	c.2731C>T	Truncating	DB-ID: MSH6_00071

MSH6_16	-ve MSH6	4	c.2976delA	Truncating	Not previously reported

MSH6_25	-ve MSH6 MSI-High	4	c.3142C>T	Nonsense substitution	Not previously reported

MSH6_3	-ve MSH6	4	c.3172+1G>T	Splice site	DB-ID: MSH6_00705*

MSH6_14	Not available	5	c.3173_3556del	Deletion	DB-ID: MSH6_00482

MSH6_13 MSH6_22	-ve MSH6 -ve MSH6	5	c.3202C>T	Truncating	DB-ID: MSH6_00487

MSH6_18	-ve MSH6	5	c.3261dupC	Frameshift	DB-ID: MSH6_00201

MSH6_12	Not available	5	c.3261delC	Frameshift/Stop	DB-ID: MSH6_00203
MSH6_9	-ve MSH6 MSI-High				

MSH6_11	-ve MSH2, +ve MSH6	5	c.3268_3274delGACCTTA	Truncating	DB-ID: MSH6_00706*

MSH6_21 MSH6_26	-ve MSH6 -ve MSH6	5	c.3312delT	Truncating	DB-ID: MSH6_00497

MSH6_15	-ve MSH6	6	c.3439-1G>T	Splice site	DB-ID: MSH6_00713*

MSH6_28	-ve MSH6	6	c.3556+3_3556+13delGAGTTTTTTGT	Splice site	DB-ID: MSH6_00661

MSH6_24	Not available	7	c.3646+2dupT	Splice site	Not previously reported

MSH6_2	MSI-High	8	c.3724del13	Frameshift/Stop	Not previously reported

26 different *MSH6 *mutations have been detected in our patient cohort. Not previously reported = not reported in the Leiden Open Variation Database (LOVD), the Mismatch Repair Genes Variant Database (Memorial University of Newfoundland) or the InSIGHT database as a HNPCC/Lynch syndrome mutation. DB-ID = Database identification number from LOVD.

^#^-ve = loss of protein expression, +ve = protein expression present

*Submitted to LOVD by our research group

Of the total 78 *MSH6 *mutation positive participants belonging to 29 families, only 21 participants (27%) had developed colorectal cancer (CRC). The average and median age of diagnosis of CRC was 48 years, ranging from 21 to 72 years. The median age of individuals who were cancer free at the time of sample collection was 44 years, ranging from 18-76, and the average age of this group was 45 years.

Cancer incidence in the *MSH6 *mutation positive families includes, in order of frequency (in how many families the cancer was observed): CRC in 23 families (79%); cancer of the endometrium in 17 families (59%); breast or prostate cancer in 7 families (24%); and ovarian cancer in 5 families (17%). Other extra colonic cancers, including lung, bladder, stomach, cervical, Hodgkin lymphoma, Non-Hodgkin lymphoma, pancreas, liver, throat, lymphoma, thyroid, leukaemia, kidney, gallbladder, brain, melanoma, acute lymphoblastic leukaemia and pituitary tumour can be seen in four or less families. In six families, cancer of an unknown site was recorded (Table 2). Extra colonic cancers were diagnosed in 2 individuals (9.5%) who had developed CRC and in 14 individuals (25%) who had not developed CRC. Table 3 provides detailed information about the extracolonic cancers in these patients. A wide spectrum of malignancies was present in the 29 *MSH6 *families and individuals with two primary tumours were observed in 14 of the 29 families (48%), see Table 2 for details.

**Table 2:** Mutation positive family members, type of cancer, age and gender, as well as other family cancers present in family members with unknown mutation status (all information collected from pedigrees) and whether the family fulfilled the Amsterdam II criteria.

Mutation positive family members: Family number - Cancer history (Age of diagnosis or age at last follow up)	Gender	Other family cancers (Age and relationship to proband) - mutation status unknown	Fulfilled Am II criteria
MSH6_1- CRC (31) MSH6_1.1- no cancer (69) MSH6_1.2- no cancer (33)	F F M	1 Endometrial (63-mother), 1 CRC + Oesophagus (mothers brother), 1 Ovarian (mothers sister), 1 Throat (mothers brother), 1 Stomach (mothers father), 1 CRC (grand fathers sister)	Yes

MSH6_2- CRC (64) MSH6_2.1- no cancer (76)	M M	1 CRC + Pancreatic (50 + 84 - mother), 1 Prostate (70-brother)	Yes

MSH6_3- CRC (50)	F	1 CRC × 2 (64 + 71 - mother), 1 CRC & Prostate (60s - mothers 1^st ^cousin)	Yes

MSH6_4- CRC(72), Endometrial (74) MSH6_4.1- CRC (37) daughter of proband MSH6_4.2- no cancer (55) MSH6_4.3- no cancer (18)	F F M F	1 CRC (70-brother), 1 Pituitary tumour (15-brothers granddaughter), 1 Breast (60s-sister who has 1 daughter with Breast and Uterine cancer (52) and 1 daughter with Brain & Lung cancer (< 64) No cancer in parents of proband - father d.76, mother died when proband was 6 months old	Yes

MSH6_5- CRC (58)	F	1 CRC (63-mother), 3 CRC (67+78+55 - maternal half siblings), 1 Endometrial (67-maternal half sister), 1 Liver (32-maternal half brother), 2 CRC (40+53-half brothers children)	Yes

MSH6_6 - no information available MSH6_6.1- no information available MSH6_6.2- no information available	F F F	No pedigree available	Unknown

MSH6_7- CRC (41)	M	1 Hodgkins Lymphoma (13-niese), 1 Bladder (fathers sister who has 2 daughters with Cervical cancer and a grandson with Lung cancer (19), 1 Prostate (fathers brother), 1 Breast (40s-fathers sister), 1 CRC (70s-grandfather who has 2 brothers with cancer of Unknown origin). No cancer in parents of proband - age 65 + 67.	Yes

MSH6_8- CRC (?) MSH6_8.1- Ovarian (49) MSH6_8.2- no cancer (59) MSH6_8.3- no cancer (29) MSH6_8.4- Appendix (14)	M F F M F	No pedigree available	Unknown

MSH6_9- Bladder (69), Endometrial (71), Thyroid (72)	F	1 Lymphoma (77-brother), 1 CRC + Prostate (81 + 75 - brother), 1 Unknown (42 - son) No cancer in parents of proband - mother d.77, father d.73	Yes

MSH6_10- CRC (21), Non-Hodgkin Lymphoma (6) MSH6_10.1- Endometrial (68), paternal grandmother MSH6_10.2- Non Hodgkin Lymphoma (28), paternal aunty MSH6_10.3- no cancer (65)	F F F F	1 CRC (70s-grandmothers uncle), 1 Breast (60-grandmothers uncle who has 1 daughter with Ovarian cancer but who is mutation negative), 1 CRC (41-grandmothers sister's son who is also mutation negative), 2 Breast cancer (53+? - grandmothers sister and grandmothers aunt, both are mutation negative)	Yes

MSH6_11- CRC (40) MSH6_11.1- no cancer (64) MSH6_11.2- no cancer (38) MSH6_11.3- no cancer (39) MSH6_11.4- no cancer (31)	M M F F F	1 CRC (63-sister), 1 Prostate (56-brother), 1 Throat (maternal grandfather) No cancer in parents - mother d.60s, dad d.70s	No

MSH6_12- no cancer (44)	M	3 Endometrial (mother and maternal aunt and grandmother), 2 Unknown (brothers - one of which is the proband somewhere else), 1 Unknown (maternal uncle)	No/Bethesda

MSH6_13- Endometrial (54) MSH6_13.1- no cancer (60) MSH6_13.2- no cancer (57) MSH6_13.3- no cancer (37) MSH6_13.4- no cancer (55) MSH6_13.5- no cancer (37) MSH6_13.6- no cancer (34) MSH6_13.7- Endometrial (56), sister	F F M F M F F F	1 CRC + Uterine (60s-mother)	Yes

MSH6_14- no cancer (48)	M	1 CRC + Prostate (64-father), 1 Endometrial (55-fathers sister), 1 CRC (62-paternal grandmother)	No

MSH6_15- CRC (54) MSH6_15.1- no cancer (43) MSH6_15.2- no cancer (67) MSH6_15.3- no cancer (31) MSH6_15.4- no cancer (70)	F F F F M	1 CRC & Ovary (47+60s - mother), 1 Endometrial/Ovarian (sister), 6 CRC (maternal uncles), 5 CRC (maternal 1^st ^cousins), 1 Leukaemia (maternal 1^st ^cousin), 1 Ovarian (maternal 1^st ^cousin)	Yes

MSH6_16- Endometrial (50), Breast (63) MSH6_16.1- Endometrial (57), Breast (61), sister	F F	1 Breast (82-mother), 1 Breast (56-1^st ^cousin), 1 Breast (40s-mothers 1^st ^cousin)	No

MSH6_17- no cancer (42)	F	1 Endometrial (41-sister), 1 Cervix (22-sister), 1 CRC (60s-father), 1 CRC + Bladder (mothers brother), 1 Breast (52-paternal grandmother), 1 Throat (79-paternal grandfather, smoker)	Yes

MSH6_18- CRC (43) MSH6_18.1- no cancer (23) MSH6_18.2- no cancer (20) MSH6_18.3- no cancer (55) MSH6_18.4- Endometrial (44), sister	F M F M F	1 Lung (d.53-father), 1 Hodgkins Lymphoma (21-niese)	No/Bethesda

MSH6_19- CRC (46+67)	M	1 Endometrial (49-mother), 1 Thyroid (44-daughter), 2 Unknown (mothers sisters)	Yes

MSH6_20- CRC (48) MSH6_20.1- no cancer 26) MSH6_20.2- no cancer (53)	F M F	1 Endometrial + CRC (54+70 - mother,1 Stomach (40s-maternal grandfather)	Yes

MSH6_21- CRC (31) MSH6_21.1- no cancer (49) MSH6_21.2- no cancer (28) MSH6_21.3- no cancer (55) MSH6_21.4- CRC (58), aunty MSH6_21.5- no cancer (37) MSH6_21.6- no cancer (60) MSH6_21.7- no cancer (38) MSH6_21.8- no cancer (29) MSH6_21.9- no cancer (33)	M F M F F F F M F F	2 CRC (39+42 - maternal uncles), 1 Lung (d.69-dad, smoker) Mother is mutation positive but with no cancer	Yes

MSH6_22- CRC (54) MSH6_22.1- no cancer (45)	M F	1 CRC (48-father), 1 Ovarian (51-fathers sister), 1 CRC (34-fathers brother who has 1 daughter with CRC (52), 1 CRC (62-fathers brother uncle), 1 Breast (67-fathers sister)	Yes

MSH6_23- Endometrial (64), Breast (70)	F	No pedigree available	Unknown

MSH6_24- CRC (50)	F	1 Renal (father), 1 Endometrial (fathers sister), 1 Gallbladder (fathers sister), 1 Stomach (dx 56 - grandfather)	Yes

MSH6_25- CRC (63)	F	1 Unknown (maternal grandfather) No cancer in parents of proband - father d.60s, mother d.80s	No

MSH6_26- CRC (66+67) MSH6_26.1- Bladder (52), brother MSH6_26.2- no cancer (68)	M M M F	2 CRC (59+59 - brothers), 1 Melanoma (brother) No cancer in parents of proband - both d.80s	No

MSH6_27- CRC (38)	M	No family history of cancer	No

MSH6_28- Endometrial (50)	F	1 Uterine (50s - mother), 1 Unknown (mothers sister), 1 Uterus (52 - maternal grandmother), 1 ALL (son), 1 Leukaemia (82 - father), 1 Prostate (paternal grandfather)	Unknown

MSH6_29- Ovarian (38)	F	No pedigree available	Unknown

**Table 3:** Details about the extra colonic cancers observed in the MSH6 patient cohort (n = 78).

Family ID	Cancer (Age of diagnosis)	Family relationship	Mutation in family- Nucleotide Change	Family fulfilled Am II criteria
MSH6_4	CRC (72) Endometrial (74)	Proband	c.2150_ 2153delTCAG	Yes

MSH6_8.1	Ovarian (49)	No pedigree available	c.458_627del	Yes

MSH6_8.4	Appendix cancer (14)	No pedigree available	c.458_627del	Yes

MSH6_9	Bladder (69) Endometrial (71) Thyroid (72)	Proband	c.3261delC	Yes

MSH6_10	CRC (21) Non-Hodgkin Lymphoma (6)	Proband	c.1_457del	Yes

MSH6_10.1	Endometrial (68)	Probands paternal grandmother	c.1_457del	Yes

MSH6_10.2	Non-Hodgkin Lymphoma (28)	Probands paternal aunty	c.1_457del	Yes

MSH6_13	Endometrial (54)	Proband	c.3202C>T	Yes

MSH6_13.7	Endometrial (56)	Probands sister	c.3202C>T	Yes

MSH6_16	Endometrial (50) Breast (63)	Proband	c.2976delA	No

MSH6_16.1	Endometrial (57) Breast (61)	Probands sister	c.2976delA	No

MSH6_18.4	Endometrial (44)	Probands sister	c.3261dupC	No/Bethesda

MSH6_23	Endometrial (64) Breast (70)	Proband	c.1404_1405delTC	Unknown (no pedigree)

MSH6_26.1	Bladder (52)	Probands brother	c.3312delT	No

MSH6_28	Endometrial (50)	Proband	c.3556+3_3556+13delGAGTTTTTTGT	Unknown

MSH6_29	Ovarian (38)	Proband	c.620del	Unknown (no pedigree)

**Figure 1:**
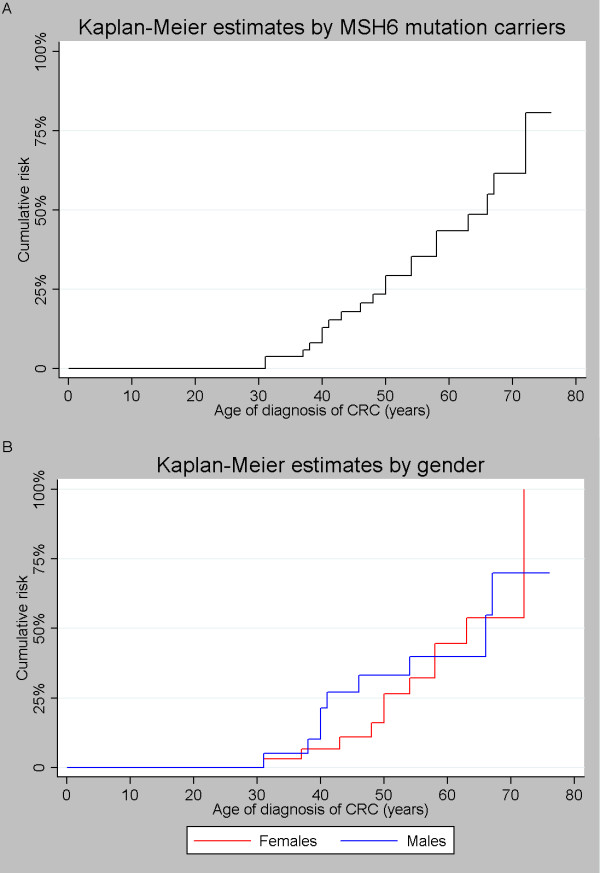
**Colorectal cancer**; cumulative risk for MSH6 mutation carriers. A) sample cohort and B) sample cohort divided by gender.

**Figure 2:**
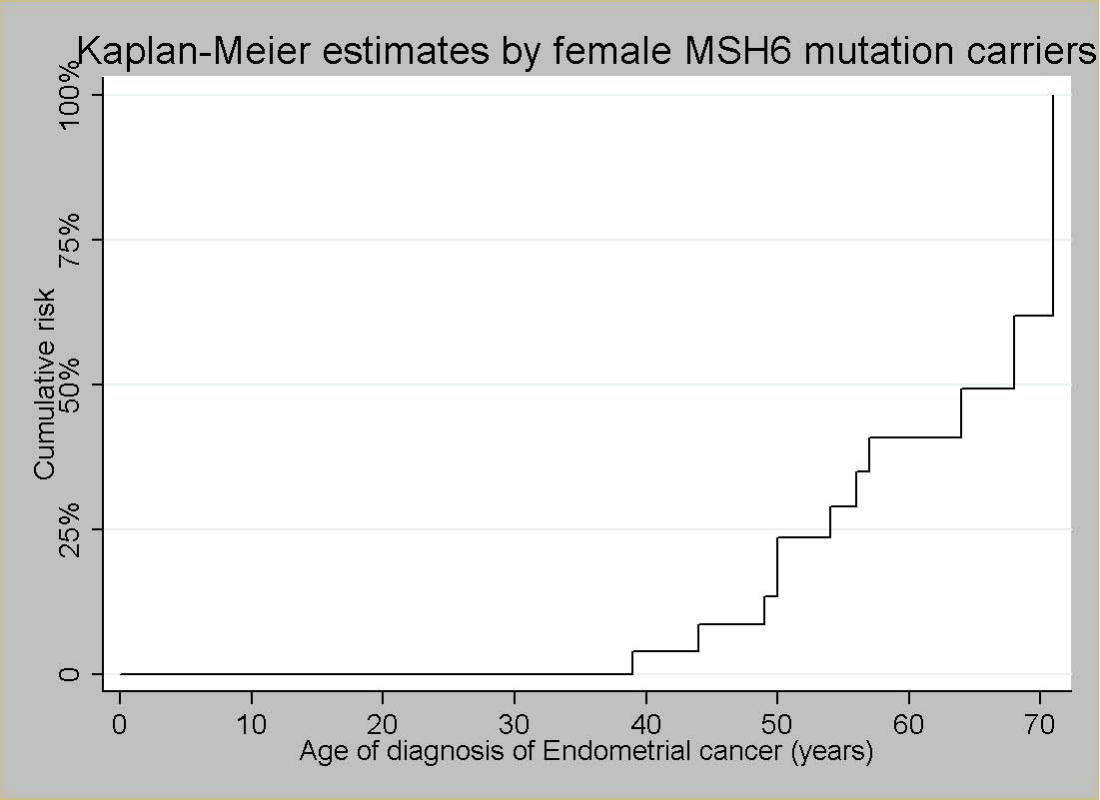
**Endometrial cancer**; cumulative risk for female MSH6 mutation carriers.

Lifetime/cumulative risk of developing colorectal and endometrial cancer are shown in Figure 1 and 2 respectively. The cumulative risk of CRC in both male and female mutation carriers at 70 years of age was 61.5% for MSH6 mutation carriers (Figure 1A), which is similar if divided by gender (Figure 1B). A difference can be seen between males and females at an earlier age with females only being at 21% risk at age 50 years, while males have a risk of ~34% at 50 years of age. The cumulative risk of endometrial cancer in woman at age 70 years was 65%.

Of the 53 female participants in this study, 10 had developed endometrial cancer and the average and median age of diagnosis was 59 and 57 years respectively, ranging from 50-74 years. In 6 of the 17 families where endometrial cancer had been recorded, there were one or more individuals who had both endometrial plus another primary tumour.

In 4 families pedigree information was not accessible and in 3 families the proband was cancer free (the proband is the first person tested from the family in our laboratory, the index person of the family might have been tested in another laboratory either national or international). For the 22 families where full family history was available, cancer was present in one of the probands' parents in 13 families.

### *PMS2 *families

A list of the 4 identified *PMS2 *mutations can be seen in Table 4. Three of the identified *PMS2 *mutations have been reported before [24-26] and one *PMS2 *mutation is a novel mutation. All mutations are considered causative and predictive testing has been offered to family members. Table 4 also lists the type of cancer and age of diagnosis in the probands of these families.

**Table 4:** *PMS2 *probands, type of cancer in proband and mutation information including; exon, nucleotide change, consequence of mutation and references.

Family ID	Cancer	Exon	Nucleotide Change	Consequence of Mutation	Reference LOVD database ID
PMS2_3	CRC (55), Renal (50)	1 8	c.1A>G +c.834_842del	Alternative DNA transcript Deletion	DB-ID: PMS2_00130

PMS2_1 PMS2_2 PMS2_5	CRC (41) No cancer (67) CRC (47)	7	c.736_741del6ins11	Truncating	DB-ID: PMS2_00187

PMS2_4	CRC (38)	7	c.746_753del	Truncating	Not previously reported

PMS2_6	CRC (60)	9	c.904_1144del	Exon deletion	DB-ID: PMS2_00195

4 different *PMS2 *mutations have been detected in our patient Cohort. Not previously reported = not reported in the Leiden Open Variation Database (LOVD), the Mismatch Repair Genes Variant Database (Memorial University of Newfoundland) or the InSIGHT database as a HNPCC/Lynch syndrome mutation. DB-ID = Database identification number from LOVD.

The 7 *PMS2 *participants include 6 probands and 1 family member. Five probands had been diagnosed with CRC at age 38, 41, 47, 55 and 60 years, one of them (CRC at 55 years) was also diagnosed with renal cancer at age 50 years. One proband has not developed CRC but was diagnosed with cancer of the small intestine at 63 years of age; the family member of this proband is currently cancer free at age 39 years.

Cancer incidence in the *PMS2 *mutation positive families includes, in order of frequency (in how many families the cancer can be seen): CRC (6 families); lung, stomach and brain cancer (2 families); endometrial + breast cancer, and ovarian + breast cancer (1 family); breast, cervical, Merkel cell and small intestine cancer (1 family).

## Discussion

The MSH6 participants included in this study are representative of all the HNPCC patients tested in New South Wales, Australia from 1997 to 2008, which we estimate is approximately half of the Australian HNPCC/Lynch syndrome families. The identified *MSH6 *mutations represent 10.3% of the pathogenic mutations identified in MMR genes in our Lynch syndrome families. This is in accordance with the expected frequencies of *MSH6 *mutations in already published material on Lynch syndrome [14,20,27,28]. We report 15 novel *MSH6 *mutations and 1 novel *PMS2 *mutations in Lynch syndrome families not listed in the Mismatch Repair Genes Variant Database (Memorial University of Newfoundland), the InSIGHT database as a Lynch syndrome mutation or the Leiden Open Variation Database (LOVD).

Families with *MSH6 *mutations have been reported to have a lower incidence of colorectal cancer (CRC) and later age of disease onset than *MLH1 *and *MSH2 *families [20], while others suggest same high lifetime risk of CRC and later age of disease onset [15,29]. In our Lynch syndrome cohort 27% had developed CRC, which is lower than expected [30]. This could be due to there being an over-representation of woman (68%) in the *MSH6 *participants, as woman have been reported to be at lower risk of CRC than men [30]. The median age of diagnosis of CRC was 48 years in the cohort studied, which is approximately 3-7 years younger than previously reported [20,27,31]. The median age for the rest of the cohort was 44 years, which is an indication of the likelihood of more people developing CRC at a later age and thereby increasing the median age of diagnosis of CRC. Exclusion of missense mutations in this study could also influence the low age of CRC observed, as cases were selected due to more severe alterations and the chance of developing CRC earlier is therefore higher. In this patient cohort the lifetime risk for CRC at age 70 years was ~61% independent of gender, which is somewhat different from the lifetime risk of MSH6 mutation carriers presented in a previous study were there was a clear difference between males and females at age 70 years [32]. The males in both studies had similar lifetime risk, but we fail to see the lower risk in woman. A meta-analysis of 5 different MSH6 mutation positive Lynch syndrome cohorts displayed a much lower lifetime risk of CRC at ~20% [33], which is an indication that our sample cohort might be too small to produce reliable lifetime risk figures.

The median age of endometrial cancer in this study was 59 years, supporting the much later onset of cancer in *MSH6 *mutation carriers [27]. Females in this patient cohort had a lifetime risk of endometrial cancer of 65% at 70 years of age, which is similar to previously reported risk figures for MSH6 mutation carriers [32] but again much higher than risk figures produced from a much larger study population [33]. Endometrial cancer was seen in 59% of the *MSH6 *families as the second most common malignancy observed. Extra colonic cancers were observed with a higher frequency in the participants who had not developed CRC compared to the participants who had developed CRC. These observations are in accordance with the cancer frequencies seen in the German Hereditary Nonpolyposis Colorectal Cancer Consortium [20]. Cancer incidence in these families was as previously reported [30] with CRC being the most common, followed by endometrial cancer. Breast and prostate cancer were observed in 23% of the *MSH6 *families, while ovarian cancer was observed in 17% of the families. Both ovarian and prostate cancer were expected to be observed in Lynch syndrome families [34] but the inclusion of breast cancer in the cancer spectrum in Lynch syndrome is controversial [35-39]. The high incidence of breast cancer in these families may genuinely reflect an increased risk of breast cancer, or it may indicate the high incidence of breast cancer in the general population (1 in 9 woman in Australia [40]).

Previously, it has been reported that patients with pathogenic *MSH6 *mutations are less frequently affected by multiple tumours [20]. This does not seem to be the case in our families as one or more individuals who had been diagnosed with two primary malignancies occurred in 14 of the 29 *MSH6 *families.

Currently the general consensus is that *MSH6 *mutations in HNPCC are under-diagnosed [25,35,41]. This is thought to be due to *MSH6 *not being routinely tested in most laboratories and that the presence of *MSH6 *mutations is under-estimated due to a more atypical presentation of disease, making the patients less likely to fulfil diagnostic criteria. This is supported by a report of an unusual high incidence of *MSH6 *mutations (21%) in Amsterdam negative families [42]. In the current study, participants were selected based on the molecular diagnosis of Lynch syndrome, nevertheless 24% of our families did not fulfil the Amsterdam II criteria. There is no routine screening for *MSH6 *in our laboratory and it is only performed when there is loss of *MSH6 *expression in the tumour (IHC) or a family history indicating *MSH6 *mutation.

*PMS2 *mutations lead to an attenuated phenotype with weaker family history and an older age of onset [15]. Communicating cancer risk to PMS2 mutation carriers and deciding which surveillance protocol is adequate for the families is a difficult task for the genetic counsellor/geneticist. In this study, only 6 *PMS2 *families were included. While this is not enough families to be able to predict a *PMS2 *phenotype, it is important that the information is publicly available so that a *PMS2 *phenotype can be made in the future.

Lynch syndrome is a complex disease with variation in disease expression influenced by both genetic and environmental factors, as evidenced by differences in genotype-phenotype within and between families with the same mutations and by ethnicity and mutated MMR gene [43-45]. To date, no worldwide genotype-phenotype correlation has been detected. Our data provides additional information to add to the genotype-phenotype spectrum for both *MSH6 *and *PMS2 *mutations. As approximately half of the clinically diagnosed HNPCC population can be classified as having Lynch syndrome (germline mutation in MMR genes), there are most likely other genomic regions that are also responsible for the disease. Future next-generation sequencing are likely to provide us with some answers by locating new genomic regions of interest, as shown by identification of the EPCAM deletion [46], but until the methodology is widely available the candidate gene approach in individual Lynch syndrome cohorts will help us in understanding the genotype-phenotype mystery.

## Competing interests

The authors declare that they have no competing interests.

## Authors' contributions

BTP: Study design; acquisition of data; analysis and interpretation of data; drafting of the manuscript; statistical analysis. MM, CG and AS: Acquisition of data. RJS: Study concept and design; critical revision of the manuscript for important intellectual content; obtained funding; study supervision. All authors read and approved the final manuscript.
